# Improvement in nurse staffing ratios according to policy changes: a prospective cohort study

**DOI:** 10.1186/s12912-024-01995-w

**Published:** 2024-05-17

**Authors:** Yunmi Kim, Kyounga Lee, Minho Jung

**Affiliations:** 1 College of Nursing, Eulji University, Seongnam-Si, Republic of Korea; 2 College of Nursing, Gachon University, Incheon, Republic of Korea; 3 Department of Nursing, Seoul National University Hospital, Seoul, Republic of Korea

**Keywords:** Nursing staff, Personnel staffing and scheduling, Policy, Reimbursement, Incentive

## Abstract

**Background:**

Since 1999, reimbursements for nursing services for inpatients have been paid differentially according to the nurse staffing ratios in Korea. However, differentiated nursing fees are insufficient for nurse staffing; thus, steps have been taken to improve the policy. This study aimed to identify the impact of a policy that changed the method of calculating nurse staffing ratios from the nurse-to-bed ratio to the nurse-to-patient ratio on improving the nurse staffing ratio in medical institutions.

**Methods:**

Data were collected from 1,339 medical institutions that continuously provided medical services from 2017 to of 2021, and a prospective cohort was used for analysis. A generalized estimating equation for longitudinal ordered logistic regression was used to identify the impact of this policy change on the nurse staffing ratios in medical institutions.

**Results:**

During the cohort study, 59.8% of the first-applied group of medical institutions and 65.6% of the second-applied group of medical institutions improved their nurse staffing ratios. However, only 22.6% of the medical institutions to which the revised calculation method was not applied improved their nurse staffing ratios. A statistically significant difference was found in the improved nurse staffing ratio depending on whether and when the revised calculation method was applied (χ^2^ = 89.830, *p* < .001). The analysis of nurse staffing ratios of medical institutions from 2017 to 2021 showed that the likelihood of improving the nurse staffing ratio increased gradually after the revised calculation method was adopted. Also,the likelihood of the nurse staffing ratio improving in the first-applied group was 1.41 times higher (odds ratio = 1.41, 95% confidence interval = 1.04–1.92) than in the non-applied group. The odds ratio for the improvement of nurse staffing ratio in the second-applied group was 2.35 (95% confidence interval = 1.76–3.14).

**Conclusions:**

Financial incentives inherent in the new policy can be regarded as the driving force behind improvements in nurse staffing ratios. The revised calculation method should be extended to all medical institutions nationwide, and the law should be revised to secure the minimum number of nurses.

## Background

In order to provide nursing services that will ensure patient safety, medical institutions should have a sufficient number of nurses [1, 2]. Some countries stipulate a maximum number of patients to be assigned to one nurse. The average nurse-to-patient ratio is less than 1:4 in Australia and 1:5 in California [1]. In medical institutions, increased levels of nurse staffing can lower patient mortality, reduce patients’ length of stay, promote patient safety, and boost patient satisfaction [3–5]. In contrast, insufficient nurse staffing has a negative impact on work-related stress, job satisfaction, desire to change careers, and instances of infection exposure [6]. Ensuring a sufficient number of nurses has a positive effect on hospital financial performance by increasing profitability and reducing expenditures, such as nurse turnover and associated costs [7, 8].

Because healthcare is a basic human need and public good, the operation of medical institutions should not be entirely entrusted to market forces [9]. For example, if a medical institution autonomously determines the level of medical personnel without government guidelines, a situation could arise in which the institution deploys the minimum number of nurses to care for the maximum number of patients to reduce nurses’ labor costs, which constitute a significant proportion of the expenditure [7, 10]. Government regulations or guidelines are required because natural monopolies and market failures may occur in medical and public health systems [11]. The government of each country, therefore, focuses on the development of policy and legislation to ensure appropriate nurse staffing in medical institutions [12, 13].

Since 1999, one of the government interventions for nurse staffing in Korea has been the nursing fee differentiation policy, which differentiates support for inpatient nursing fees based on nurse staffing grades [14]. The nurse staffing grade of the Korean policy refers to the nurse staffing ratio. Nurse staffing grades were measured by the ratio of nurses to beds (or patients) according to the type of hospital and department. The nurse staffing grades were classified from grade 1 (the best) to grade 7 (the worst) and were calculated by dividing the average number of beds (or patients) over the past three months (as of the 15th of each month) by the average number of nurses over the same period. The number of nurses applied to the nurse staffing grades excludes nurses who are not engaged in patient care and includes only nurses who actually provide patient care in wards and intensive care units (ICUs). When calculating nurse staffing grades, the severity of patients’ conditions or the acuity of illness was not adjusted within the same ward or ICU, but different grading standards were applied to general wards and ICUs, respectively. For example, in the case of a general ward, grade 1 (the best) means that the number of beds (or patients) per nurse must be less than 2.5, but in the case of an ICU, the number must be less than 0.5. Further, even in the same general ward, in the case of a tertiary hospital that cares for patients with high-disease severity, grade 1 means that the number of beds (or patients) per nurse is less than 2.0 [15]. Medical institutions are required to report their nurse staffing grades to the government every quarter.

The nursing fee differentiation policy was introduced in Korea medical institutions lacking sufficient nurse staffing levels to address the issue of the deteriorating quality of nursing services for inpatients, such as partial nursing service omission or delegation of nursing services to guardians or caregivers [16]. The nursing fee differentiation policy is a financial incentive reimbursement system, in which inpatient nursing fees increase by a certain percentage as the nursing staff level increases.

Korea has the National Health Insurance (NHI) healthcare system operated by the government insurer, NHI Service (NHIS) [17]. When inpatients use the service of a hospital, they pay 5–20% of the total medical expenses directly to the hospital as a co-payment, and the hospital bills the NHIS for the remaining amount and is reimbursed [18]. All citizens pay approximately 7% of their monthly income as insurance contributions every month. In Korea, a fee-for-service model basically determines the cost of healthcare services, and hospitalization fees are charged separately per inpatient per day. The inpatient nursing fee constitutes a certain percentage of hospitalization fees and the inpatient nursing fee is affected by nurse staffing grades. If the hospital’s nurse staffing grade is 1, the hospital will reimburse all inpatient nursing fees and certain extra financial incentives. In the case of hospitals with a nurse staffing grade of 7, reduced inpatient nursing fees will be reimbursed [19].

This nursing fee differentiation policy was initially applied to general wards (Table 1) and then expanded to ICUs by the Korean government. However, contrary to the policy intention that medical institutions would voluntarily increase nurse staffing levels, small- and medium-sized regional hospitals renounced financial incentives, received only minimum base inpatient nursing fees, and did not hire additional nurses. Accordingly, from 2007, the Korean government adopted a disincentive policy that reduced inpatient nursing fees by 2–5% for medical institutions that did not maintain basic nurse staffing levels [14].

**Table 1:** The criteria for the nursing fee differentiation policy by nursing staffing ratios of general wards

Classification	Number of patients (or beds) per nurse	Inpatient nursing fees	
		General hospitals	Hospitals
Grade 1	< 2.5	Increase of 10% from grade 2	Increase of 10% from grade 2
Grade 2	≤ 2.5 and < 3.0	Increase of 10% from grade 3	Increase of 10% from grade 3
Grade 3	≤ 3.0 and < 3.5	Increase of 15% from grade 4	Increase of 10% from grade 4
Grade 4	≤ 3.5 and < 4.0	Increase of 10% from grade 5	Increase of 10% from grade 5
Grade 5	≤ 4.0 and < 4.5	Increase of 10% from grade 6	Increase of 20% from grade 6
Grade 6	≤ 4.5 and < 6.0	Reference grade	Reference grade
Grade 7	≥ 6.0	Reduction of 2–5% of grade 6	Reduction of 2–5% of grade 6

Nevertheless, the nursing fee differentiation policy had little effect as an incentive on the improvement of nurse staffing levels, and the method used to calculate nurse staffing grades was identified as one of the causes of the problem. In early years, the nursing fee differentiation policy used the nurse-to-bed ratio to calculate nurse staffing grade. Since this calculation method is based on the number of beds regardless of the number of patients for whom nurses actually cared, a problem emerged—namely, in medical institutions with low bed utilization, there was no major change in the nurse staffing grade, even if the quality of care was improved by hiring additional nurses, and the cost of hiring additional nurses increased without any corresponding increase in reimbursement [20].

Finding nurses for small- and medium-sized regional hospitals has become difficult owing to expectation of high wages among new nurses who enter the medical field, their preference for working in large hospitals, and their tendency to avoid working in less populous regions. Therefore, the need to improve the existing calculation method for the nurse staffing grade emerged [21, 22]. Accordingly, in 2017, the Korean government proposed a revised plan to change the method of calculating nurse staffing grades from the nurse-to-bed ratio to the nurse-to-patient ratio [21].

As of April 1, 2018, this revised method of calculation was adopted in medical institutions in small- and medium-sized cities and rural areas, as well as in some medical institutions established in accordance with certain legislation (first stage of application). The revised calculation method aimed to resolve the disadvantages of the low bed utilization rate in medical institutions in small- and medium-sized cities and rural areas and to enhance the treatment of nurses by providing additional nursing fee compensation (revenue) for hospitals with higher nurse staffing grades [23]. Through this reform, the Korean government attempted to solve the difficulties of medical institutions in securing nurses in small- and medium-sized cities and rural areas. In 2020, all nationwide medical institutions were subject to the revised calculation method for nurse staffing grades, except for tertiary medical institutions (45 nationwide as of 2021) and medical institutions located in the capital, Seoul (second stage of application). Tertiary medical institutions and institutions located in the capital were excluded because they had a high bed utilization rates and less difficulty securing nurses.

Securing a sufficient number of medical personnel is recognized as a key factor in operating medical institutions and navigating the healthcare system [24]. Nurses are important personnel in medical institutions. Incentive policies and regulations at the government level are essential for securing and retaining the sufficient number of nurses to meet patients’ needs in medical institutions in small- and medium-sized cities and rural areas, which face major difficulties providing sufficient nurses [14]. In 2004, California introduced legislation mandating a minimum nurse-to-patient ratio, which increased the level of nurse staffing in medical institutions and direct nursing time [25]. In Australia, medical institutions that implemented mandatory regulations for a minimum nurse-to-patient ratio acquired more nurses than those that did not implement these regulations. They also reported improvements in patients’ health indicators such as mortality, readmission rate, and the length of hospital stay [12].

The nursing fee differentiation policy, based on nurse staffing grades calculated using the nurse-to-bed ratio, improved nurse staffing levels in many large medical institutions in large cities [26], whereas the effect of this policy on local small- and medium-sized hospitals was insignificant. In 2018 and 2020, the Ministry of Health and Welfare switched the method for calculating nurse staffing grades to the nurse-to-patient ratio to improve the nursing fee differentiation policy, which had stagnated as a financial incentive to secure nurses. However, in Korea, the period of nursing education an individual required to become a nurse had been unified into a four-year bachelor's degree program in 2011. Thus, as of 2024, all universities and colleges offered nursing education as a 4-year bachelor's program, instead of dividing it into a 3-year diploma program and a 4-year bachelor's program [27]. Further, unlike foreign cases with various types of nursing personnel, the main nursing personnel in Korean general hospitals are all registered nurses (RNs). Therefore, the nursing-fee differentiation policy is also based on the number of RNs per patient (or bed). Therefore, following the direction of Korea’s nursing education, this policy aimed to reduce the number of patients per nurse by increasing the number of nurses caring for patients without considering their educational background.

The study aimed to examine the impact of the nursing fee differentiation policy, which changed the nurse-to-bed ratio to the nurse-to-patient ratio, on the improvement of nurse staffing grades (referring to ratio) in medical institutions.

## Methods

### Study design

This prospective cohort study used national-level secondary data to assess the impact of the revised nursing fee differentiation policy based on the nurse-to-patient ratio on changes in nurse staffing grades in medical institutions. However, since nurse staffing grade is a term used in Korea, nurse staffing ratio replaced it to improve international understanding. In other words, the nurse staffing ratio described in this study refers to the Korean nurse-staffing grade, which ranges from one to seven. This study was designed, analyzed, and described in accordance with STROBE guidelines.

### Participants and data sources

This study began by building a prospective cohort in the second quarter of 2017 and completed the cohort construction in the second quarter of 2021. We collected data on the nurse staffing ratio of general ward; the type of hospital and establishment; location; and the number of beds, physicians, and medical equipment for all 1,806 hospital-level medical institutions in Korea. These data were collected from the website of Health Insurance Review & Assessment (HIRA) Service via the “Find hospitals and pharmacies” menu [28]. The HIRA reviews the claims and assesses the quality of health care services. The HIRA sets the scope and standards of services covered by NHI, efficiently manages healthcare resources, and evaluates the cost and quality of healthcare services. All medical institutions were required to submit claims and hospital information to the HIRA. Using this information, the HIRA provides the latest updated basic hospital information (operation hours, the number of beds, medical departments, number of physicians, emergency room operations, medical equipment, and nurse staffing ratio), hospital evaluation information (results of evaluating hospital medical services, including surgery, disease, and drug use from medical/pharmaceutical and cost-effective aspects), and medical expenses information. All citizens had real-time access to data on the HIRA website. This study used these data to analyze changes in nurse staffing ratios according to changes in nursing fee differentiation policy.

The following medical institutions were excluded from our study: 1) those that focused on special patients such as military hospitals, police hospitals, and national Hansen’s disease hospitals, because these hospitals operate for special purposes without any medical profits unlike general hospitals. They receive medical expenses from the government, military, and civil servant organizations, rather than from patients; therefore, financial-incentive policies may not generally affect them. Further, the nursing staff at military hospitals consists of nursing officers who are military personnel; so there is the difference of the supply and demand of nursing staff at general hospitals; 2) institutions that carry descriptions, such as “nursing home,” “rehabilitation,” “geriatric,” and “psychiatric,” in their names, or those that functioned minimally as acute care hospitals by providing internal surgery because the number of beds in closed psychiatric wards accounted for more than 50% of the number of beds in general wards; 3) those that had changed their hospital type to clinics or convalescent hospitals; 4) those that were newly established or closed during the study period; and 5) tertiary hospitals. Even if the name of a medical institution changed during the prospective cohort period, medical institutions located at the same physical address were classified into the same cohort. For the final analysis, 1,339 hospital-level medical institutions that satisfied the research criteria and had provided medical services continuously for five years from 2017 to 2021 were chosen.

### Measurement

#### Outcome variable

To determine the effect of the application of the revised calculation method of the nursing fee differentiation policy on the improvement of medical institutions’ the nurse staffing ratios, this study used the nurse staffing ratio of the general ward of each medical institution in the second quarter of each year from 2017 to 2021 as an outcome variable. Based on the grading classification as part of the nursing fee differentiation policy, nurse staffing ratios of the general wards were classified according to the number of patients (number of beds before the revision) per nurse in the general wards.

Nurse staffing ratios were calculated by dividing the average number of patients (or beds) for three months from April to June each year by the average number of nurses during the same period and had values ranging from grade 1 to grade 7.

The specific criteria for each grade are shown in Table 1; using grade 6 as the reference point, the inpatient nursing fee increases by 10% to 20% of the immediately lower grade in grades 1 to 5, and decreases by 2% to 5% depending on the location of the medical institution in grade 7. Since grade 1 was the highest grade and grade 7 was the lowest grade for nurse staffing ratio, this grade was reverse-coded and analyzed for ordered logistic regression.

#### Other variables

Medical institutions included in the study were divided into three categories based on when the revised calculation method for the nurse staffing ratio was implemented: the first-applied group (medical institutions to which the revised calculation method was applied from 2018), second-applied group (institutions to which the revised calculation method was applied from 2020), and not applied group (medical institutions excluded from the revised calculation method). Medical institutions were divided into general hospitals and hospitals. According to Korean Medical Law, a hospital is a medical institution with at least 30 beds and is designated as a general hospital if specific criteria are satisfied, such as having more than 100 inpatient beds, establishing more than seven departments, and having a specialist in each department. The type of establishment of medical institutions was divided into public and private hospitals, and the total number of beds in medical institutions was divided into four categories: fewer than 50, 50 to 99, 100 to 199, and 200 or more. Based on the ratio of non-insurance-covered beds that are not covered by NHI, for which the patient pays 100% of the hospital bed charges, hospitals were classified into less than 5%, 5% to less than 10%, 10% to less than 15%, and 15% or more. The physician staffing level was measured as the number of physicians (including specialists) per 100 beds and divided into four categories: fewer than 5, 5 to fewer than 10, 10 to fewer than 15, and 15 or more physicians. The level of medical equipment acquisition was measured as the number of magnetic resonance imaging (MRI) devices per 100 beds (at least one, more than zero but less than one, and zero).

### Data analysis

The distribution of nurse staffing ratios and the characteristics of medical institutions in the study were described using descriptive statistics, such as frequencies and percentages. The distribution and changes in nurse staffing ratios according to the characteristics of the medical institutions were analyzed using the chi-square test or Fisher's exact test. Changes in the distribution of nurse staffing ratios according to the characteristics of medical institutions during the cohort period were analyzed by comparing the nurse staffing ratios in 2017 with those in 2021. Longitudinal data analysis was performed to examine the effect of the revised calculation method for the nurse staffing ratio (treatment effect) on the overall changes in nurse staffing ratios of each medical institution from 2017 to 2021(time effect). As the outcome variable, the nurse staffing ratio is an ordinal variable ranging from 1 to 7, a multivariable generalized estimating equation model was used to conduct ordered logistic regression. Model 1 reflected only the application period of the revised nurse staffing ratio as an independent variable; Model 2 reflected the application period and year, and Model 3 reflected the application period and all control variables.

## Results

### Distribution of nurse staffing ratios and general characteristics of the participating medical institutions

Among 1,339 medical institutions studied, 664 (49.6%) were part of the first-applied group of medical institutions that used the revised nurse staffing ratio (which began in 2018), and 476 (35.6%) were part of the second-applied group of medical institutions, which began in 2020. For 199 (14.9%) medical institutions, the existing nurse staffing ratio, which is based on the number of beds, was applied. Table 2 shows the distribution of nurse staffing ratios and the general characteristics of the 1,339 participating medical institutions in the second quarter of 2021. Regarding institutions to which the revised nurse staffing ratio was not applied (the non-applied group of medical institutions), most were in grade 7 (57.3%). In the first-applied group, grade 7 was allotted to 35.8% of institutions and grade 1 was allotted to 33.3% of institutions. In the second-applied group, grade 1 was allotted to the most institutions, at 40.8% (χ^2^ = 89.830, *p* < 0.001). Significant differences were observed in nurse staffing ratios by the type of medical institution (χ^2^ = 166.673, *p* < 0.001). The nurse staffing ratios of 280 general hospitals (20.9%) were grade 1 (57.5%) followed by grade 2 (13.2%), whereas the nurse staffing ratios of 1,059 hospitals (79.1%) were grade 7 (44.6%), followed by grade 1 (25.4%). In terms of establishment type, private institutions (1,282, 95.7%) had the highest percentage of grade 7 (38.5%), while public institutions had the highest percentage of grade 1 (75.4%). Nurse staffing ratios showed a significant difference according to the number of beds in medical institutions (χ^2^ = 152.992, *p* < 0.001). In medical institutions with a high proportion of non-insurance-covered beds, the ratio of grade 1 was higher (χ^2^ = 99.301, *p* < 0.001). There was a significant difference in the distribution of nurse staffing ratios according to the number of physicians per 100 beds (χ^2^ = 206.088 *p* < 0.001). Medical institutions with fewer than 5 physicians per 100 beds had the highest proportion of grade 7 (64.4%). Among medical institutions with fewer than 1 MRI device per 100 beds, grade 1 was the most prevalent (40.9%) (χ^2^ = 103.955 *p* < 0.001).

**Table 2:** General characteristics of subject medical institutions (2021)

Variable	Category	Total n (%)	Nurse staffing ratio in 2021 n (%)							χ^2^ (*p*)
			**Grade 1**	**Grade 2**	**Grade 3**	**Grade 4**	**Grade 5**	**Grade 6**	**Grade 7**	
Revised nurse staffing grade applied	Second group (2020)	476 (35.6)	194 (40.8)	31 (6.5)	34 (7.1)	21 (4.4)	20 (4.2)	31 (6.5)	145 (30.5)	89.830 (< .001)
	First group (2018)	664 (49.6)	221 (33.3)	58 (8.7)	40 (6.0)	35 (5.3)	21 (3.2)	51 (7.7)	238 (35.8)	
	Not applied	199 (14.9)	15 (7.5)	15 (7.5)	16 (8.0)	5 (2.5)	9 (4.5)	25 (12.6)	114 (57.3)	
Hospital type	Hospital	1,059 (79.1)	269 (25.4)	67 (6.3)	68 (6.4)	47 (4.5)	44 (4.2)	92 (8.7)	472 (44.6)	166.673 (< .001)
	General hospital	280 (20.9)	161 (57.5)	37 (13.2)	22 (7.9)	14 (5.0)	6 (2.1)	15 (5.4)	25 (8.9)	
Establishment type	Public	57(4.3)	43 (75.4)	6 (10.5)	2 (3.5)	2 (3.5)	0 (0.0)	1 (1.8)	3 (5.3)	57.835^*^ (< .001)
	Private	1,282 (95.7)	387 (30.2)	98 (7.6)	88 (6.9)	59 (4.6)	50 (3.9)	106 (8.3)	494 (38.5)	
Total number of beds	< 50	268 (20.0)	95 (35.5)	6 (2.2)	18 (6.7)	4 (1.5)	8 (3.0)	20 (7.5)	117 (43.7)	152.992 (< .001)
	50–99	450 (33.6)	119 (26.4)	28 (6.2)	28 (6.2)	17 (3.8)	15 (3.3)	43 (9.6)	200 (44.4)	
	100–199	292 (21.8)	59 (20.2)	26 (9.0)	14 (4.8)	20 (6.9)	14 (4.8)	25 (8.6)	134 (45.9)	
	≥ 200	329 (24.6)	157 (47.7)	44 (13.4)	30 (9.1)	20 (6.1)	13 (4.0)	19 (5.8)	46 (14.0)	
Non insurance-covered beds	< 5%	231 (17.3)	33 (14.3)	13 (5.6)	13 (5.6)	13 (5.6)	7 (3.0)	22 (9.6)	130 (56.3)	99.301 (< .001)
	≥ 5 and < 10%	492 (36.7)	140 (28.5)	46 (9.4)	44 (8.9)	25 (5.1)	20 (4.1)	40 (8.1)	177 (36.0)	
	≥ 10 and < 15%	269 (20.1)	100 (37.2)	22 (8.2)	14 (5.2)	17 (6.3)	15 (5.6)	20 (7.4)	81 (30.1)	
	≥ 15%	347 (25.9)	157 (45.2)	23 (6.6)	19 (5.5)	6 (1.7)	8 (2.3)	25 (7.2)	109 (31.4)	
Number of physicians per 100 beds	< 5	292 (21.8)	29 (9.9)	6 (2.1)	14 (4.8)	11 (3.8)	18 (6.2)	26 (8.9)	188 (64.4)	206.088 (< .001)
	≥ 5 and < 10	440 (32.9)	124 (28.2)	40 (9.1)	40 (9.1)	31 (7.1)	14 (3.2)	47 (10.7)	144 (32.7)	
	≥ 10 and < 15	300 (22.4)	137 (45.7)	32 (10.7)	19 (6.3)	11 (3.7)	10 (3.3)	15 (5.0)	76 (25.3)	
	≥ 15	307 (22.9)	140 (45.6)	26 (8.5)	17 (5.5)	8 (2.6)	8 (2.6)	19 (6.2)	89 (29.0)	
Number of MRI per 100 beds	0	555 (41.5)	154 (27.8)	20 (3.6)	26 (4.7)	15 (2.7)	22 (4.0)	43 (7.8)	275 (49.6)	103.955 (< .001)
	> 0 and < 1	450 (33.6)	184 (40.9)	53 (11.8)	33 (7.3)	30 (6.7)	14 (3.1)	32 (7.1)	104 (23.1)	
	≥ 1	334 (24.9)	92 (27.5)	31 (9.3)	31 (9.3)	16 (4.8)	14 (4.2)	32 (9.6)	118 (35.3)	
Total		1,339 (100.0)	430 (32.1)	104 (7.8)	90 (6.7)	61 (4.6)	50 (3.7)	107 (8.0)	497 (37.1)	-

^*^Fisher’s exact test

### Annual changes in nurse staffing ratios

**Fig. 1:**
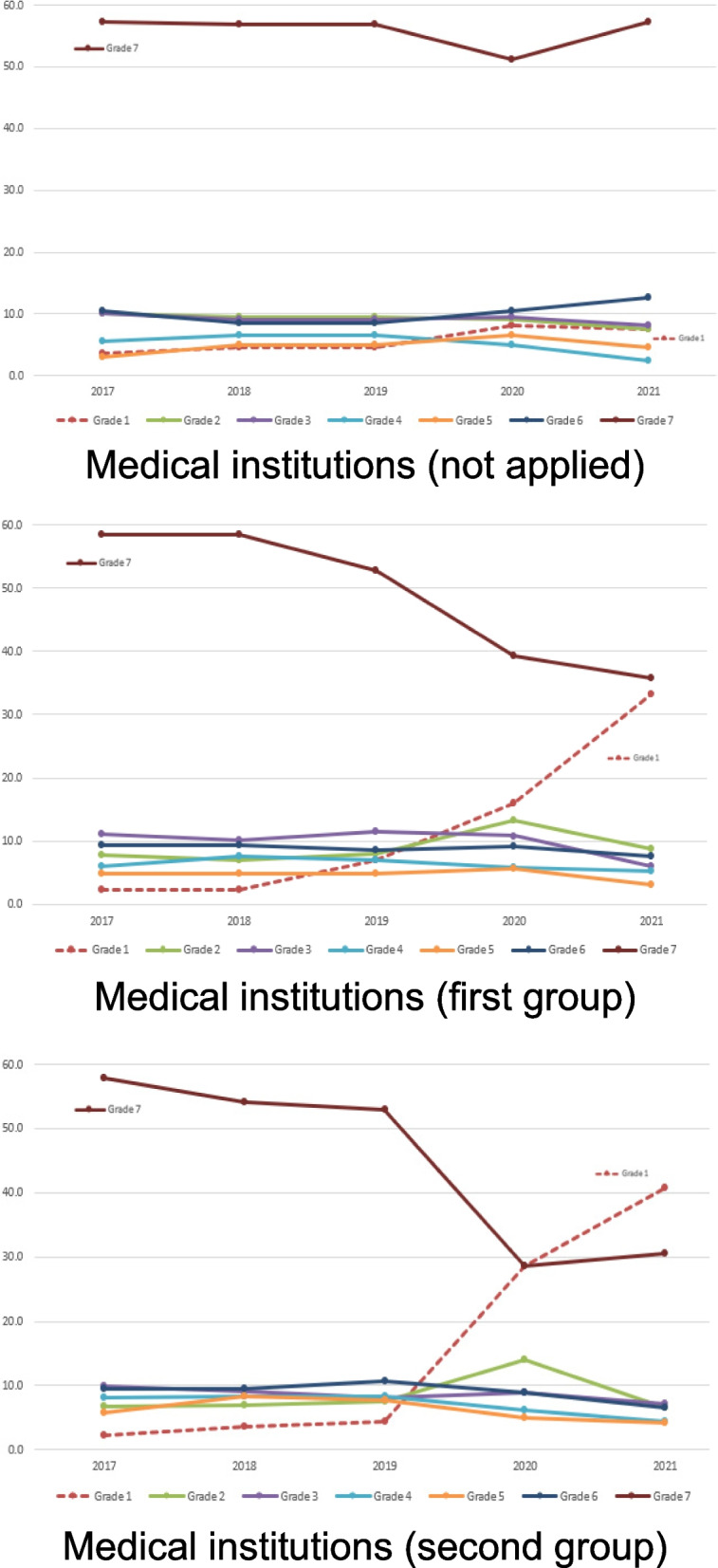
Annual changes in nurse staffing ratios

Figure 1 and Table 3 show annual changes in nurse staffing ratios from 2017 to 2021 in the medical institutions included in this study. The non-applied group showed little annual changes in nurse staffing ratios, but the proportion of grade 7 institutions decreased, and the proportion of grade 1 institutions dramatically increased in the first-applied group and second-applied group. In 2021, the proportion of grade 1cases increased to 40.8%, which is higher than the proportion of grade 7 cases (30.5%) in second-applied group.

**Table 3:** Annual changes in nurse staffing ratios

Year/Grade		2017	2018	2019	2020	2021
Not applied	1	3.5	4.5	4.5	8.0	7.5
	2	10.1	9.6	9.6	9.1	7.5
	3	10.1	9.1	9.1	9.6	8.0
	4	5.5	6.5	6.5	5.0	2.5
	5	3.0	5.0	5.0	6.5	4.5
	6	10.6	8.5	8.5	10.6	12.6
	7	57.3	56.8	56.8	51.3	57.3
First group	1	2.4	2.4	7.1	16.0	33.3
	2	7.8	7.1	8.0	13.3	8.7
	3	11.1	10.2	11.6	10.8	6.0
	4	6.0	7.5	7.1	5.9	5.3
	5	4.8	5.0	4.8	5.6	3.2
	6	9.3	9.3	8.6	9.2	7.7
	7	58.4	58.4	52.9	39.3	35.8
Second group	1	2.3	3.6	4.4	28.6	40.8
	2	6.7	6.9	7.6	14.1	6.5
	3	9.9	9.0	8.2	8.8	7.1
	4	8.2	8.4	8.4	6.1	4.4
	5	5.7	8.4	7.8	5.0	4.2
	6	9.5	9.5	10.7	8.8	6.5
	7	57.8	54.2	52.9	28.6	30.5

### Changes in nurse staffing ratios with the application of the revised policy

Depending on whether and when the revised nurse staffing ratios were implemented, a significant difference in the distribution of medical institutions with improved nurse staffing ratios was observed (χ^2^ = 141.26, *p* < 0.001) (Table 4). During the cohort period, 754 institutions (56.3%) showed improved nurse staffing ratios. Among the first-applied group, 59.8% of the institutions improved their nurse staffing ratios, and among the second-applied group, 65.6% of the institutions improved their nurse staffing ratios. However, only 22.6% of the non-applied group improved their nurse staffing ratios. Based on the characteristics of the medical institutions, 78.2% of general hospitals had improved nurse staffing ratios. The ratio of institutions with improved nurse staffing ratios was higher in public institutions than in private ones, and in hospitals with a high total number of beds and a high percentage of non-insurance-covered beds.

**Table 4:** Changes in nurse staffing ratios with the application of the revised calculation method based on the number of patients

**Classification**		**2017 → 2021 Changes in nurse staffing ratios** **n (%)**				**χ**^**2**^ (*p*)
		**Improved**	**Sustained**	**Deteriorated**	**Total**	
Number of medical institutions		754(56.3)	498(37.2)	87 (6.5)	1,339 (100.0)	-
Revised nurse staffing grade applied	Second group (2020)	312(65.6)	130(27.3)	34(7.1)	476 (100.0)	141.26 (< .001)
	First group (2018)	397(59.8)	249(37.5)	18(2.7)	664 (100.0)	
	Not applied	45(22.6)	119(59.8)	35(17.6)	199 (100.0)	
Hospital type	Hospital	535(50.5)	451(42.6)	73(6.9)	1,059 (100.0)	71.02 (< .001)
	General hospital	219(78.2)	47(16.8)	14(5.0)	280 (100.0)	
Establishment type	Public	45(79.0)	10(17.5)	2(3.5)	57 (100.0)	12.42 (.002)
	Private	709(55.3)	488(38.1)	85(6.6)	1,282 (100.0)	
Total number of beds	< 50	137(51.1)	116(43.3)	15(5.6)	268 (100.0)	58.80 (< .001)
	50–99	224(49.8)	184(40.9)	42(9.3)	450 (100.0)	
	100–199	151(51.7)	125(42.8)	16(5.5)	292 (100.0)	
	≥ 200	242(73.6)	73(22.2)	14(4.3)	329 (100.0)	
Non-insurance-covered bed	< 5%	85(36.8)	122(52.8)	24(10.4)	231 (100.0)	54.84 (< .001)
	≥ 5 and < 10%	283(57.5)	168(34.2)	41(8.3)	492 (100.0)	
	≥ 10 and < 15%	169(62.8)	92(34.2)	8(3.0)	269 (100.0)	
	≥ 15%	217(62.5)	116(33.4)	14(4.0)	347 (100.0)	
Number of physicians per 100 beds	< 5	97(33.2)	189(64.7)	6(2.1)	292 (100.0)	136.74 (< .001)
	≥ 5 and < 10	280(63.6)	138(31.4)	22(5.0)	440 (100.0)	
	≥ 10 and < 15	199(66.3)	73(24.3)	28(9.3)	300 (100.0)	
	≥ 15	178(58.0)	98(31.9)	31(10.1)	307 (100.0)	
Number of MRI per 100 beds	0	262(47.2)	275(49.6)	18(3.2)	555 (100.0)	117.43 (< .001)
	> 0 and < 1	304(67.6)	130(28.9)	16(3.6)	450 (100.0)	
	≥ 1	188(56.3)	93(27.8)	53(15.9)	334 (100.0)	

### The impact of the revised policy on changes in medical institutions’ nurse staffing ratios

Table 5 shows the impact of the revised policy on changes in nurse staffing ratios. In Model 1, the likelihood of an improved nurse staffing ratio was 1.40 times (odds ratio [OR] = 1.40, 95% confidence interval [CI] = 1.07–1.83) higher in the first-applied group and 1.65 times (OR = 1.65, 95% CI = 1.26–2.17) higher in the second-applied group compared to the non-applied group. In Model 2, which was adjusted for the year, the likelihood of nurse staffing ratio improvement was 1.48 times higher in the first-applied group (OR = 1.48, 95% CI = 1.12–1.96), and 1.78 times higher in the second-applied group (OR = 1.78, 95% CI = 1.34–2.37) compared to the non-applied group. In Model 3, which controlled for all covariates that could affect changes in nurse staffing ratio, the first-applied group was 1.41 times more likely to show nurse staffing ratio improvement (OR = 1.41, 95% CI = 1.04–1.92), and the second-applied group was 2.35 times more likely (OR = 2.35, 95% CI = 1.76–3.14).

**Table 5:** Effects of applying the revised policy on changes in nurse staffing ratios

Variable	Category	Model 1				Model 2				Model 3			
		OR	95% CI		*p*	OR	95% CI		*p*	OR	95% CI		*p*
Revised nurse staffing ratio applied (ref = not applied)	Second group (2020)	1.65	1.26	2.17	< .001	1.78	1.34	2.37	< .001	2.35	1.76	3.14	< .001
	First group (2018)	1.40	1.07	1.83	.013	1.48	1.12	1.96	.007	1.41	1.04	1.92	.028
Year (ref = 2017)	2018					1.04	1.00	1.08	0.031	1.05	1.00	1.10	.047
	2019					1.22	1.16	1.28	< .001	1.26	1.18	1.34	< .0001
	2020					2.67	2.46	2.91	< .001	3.51	3.17	3.88	< .001
	2021					3.51	3.17	3.89	< .001	5.15	4.54	5.84	< .001
Hospital type (ref = hospital)	General hospital									2.41	1.67	3.47	< .001
Establishment type (ref = public)	Private									0.46	0.32	0.66	< .001
Total number of beds (ref = < 50)	50–99									1.22	0.92	1.63	.169
	100–199									1.77	1.23	2.54	.002
	≥ 200									3.72	2.38	5.83	< .001
Non-insurance-covered beds (ref = < 5%)	≥ 5 and < 10%									1.63	1.24	2.14	.000
	≥ 10 and < 15%									1.57	1.15	2.13	.004
	≥ 15%									2.94	2.09	4.13	< .001
Number of physicians per 100 beds (ref = < 5)	≥ 5 and < 10									2.91	2.13	3.98	< .001
	≥ 10 and < 15									5.05	3.58	7.11	< .001
	≥ 15									6.75	4.70	9.70	< .001
Number of MRI per 100 beds (ref = 0)	> 0 and < 1									0.86	0.59	1.26	.441
	≥ 1									2.38	1.79	3.17	< .001

## Discussion

### Impact of the revised policy on the improvement of nurse staffing ratios

The method of calculating nurse staffing ratios is the basis for the nursing fee differentiation policy and reimbursement for inpatient nursing services in Korea. This study analyzed 1,339 medical institutions from 2017 to 2021 to determine the effects of the revised calculation method for the nurse staffing ratio (i.e., a change from the nurse-to-bed ratio to the nurse-to-patient ratio) on improvements in nurse staffing ratios. In 1999, when the nursing fee differentiation policy was first introduced, there were no general hospitals or hospitals with grade 1. However, 87.0% of general hospitals and 97.5% of hospitals were grade 6, which indicated that most medical institutions had low nurse staffing levels [26]. When analyzing the nurse staffing ratios of medical institutions in the second quarter of 2021, 57.5% of general hospitals and 25.4% of hospitals were classified as grade 1, demonstrating a significant improvement in nurse staffing levels at medical institutions compared to the early stages of policy introduction. After the revised calculation method for the nurse staffing ratio was introduced, financial incentives inherent in the new method can be regarded as the driving force behind the improvement in nurse staffing ratios. As the criterion for calculating the nurse staffing ratio shifts from the number of beds to that of patients, a hospital-level institution can earn approximately $3,000 per nurse per year if the nurse staffing ratio is improved by one grade [29]. This financial reward may facilitated medical institutions’ decision to hire additional nurses.

Following the introduction of the revised calculation method for the nurse staffing ratio, the Ministry of Health and Welfare of Korea released “Measures for Improving the Working Environment and Treatment of Nurses” and proposed guidelines. According to these guidelines, medical institutions to which the revised calculation method for the nurse staffing ratio has been applied should use more than 70% of the additional revenue (inpatient nursing fees) obtained by system reform to increase employment and improve working conditions [23]. In other words, the revised calculation method for the nurse staffing ratio aimed to improve nurses’ working environment by converting part-time nurses into full-time employees, hiring additional nurses, and increasing their wages. The exclusion of tertiary hospitals and medical institutions in the capital with relatively superior working conditions from the revised nurse staffing ratio system was intended to provide financial incentives to medical institutions with insufficient nurse staffing levels, wages, and working environments to aggressively improve nursing conditions. This study confirmed that the first-applied group and second-applied group, to which the revised nurse staffing ratio was applied, improved their nurse staffing ratios more aggressively than the non-applied group (i.e., tertiary hospitals and medical institutions located in capital), which can be evaluated as indicating that the primary purpose of the government policy was successfully achieved. In future, it will be necessary to evaluate whether nurses’ wages, employment arrangements, and working environment have improved in medical institutions with improved nursing ratios. Conducting such an evaluation would make it possible to determine whether the revised nurse staffing ratio policy ultimately succeeded.

### Variations in responses to the revised calculation method for the nurse staffing ratio

Although the nurse staffing ratios of many medical institutions improved after applying the revised calculation method, the odds ratio for the improvement in nurse staffing ratio in general hospitals was 2.41 compared to hospitals. As of 2021, 44.6% of hospitals were still at grade 7, which is quite high. In addition to the disincentive policy that has reduced 2–5% of the reference grade (grade 6) inpatient nursing fees for medical institutions with grade 7, the lowest level of nurse staffing, a positive financial incentive system based on the revised nurse staffing ratio was introduced in 2018 and 2020. However, it should be highlighted that a significant number of hospitals are not receptive to improving nurse staffing levels.

Compared to the non-applied group, the OR for improvement in the nurse staffing ratio in the first-applied group was 1.41 (95% CI = 1.01–1.92), and that of the second-applied group was 2.35 (95% CI = 1.76–3.14), indicating a higher probability of nurse staffing ratio improvement in the second-applied group. Most institutions in the first-applied group were located in small- and medium-sized cities and rural areas, while the institutions in the second-applied group were located in metropolitan cities and districts. There are several reasons why the second-applied group responded more actively to the revised nurse staffing ratio than the first-applied group. First, it is common that innovation and new medical technologies in medical institutions spread in an S-shaped curve [30], and the medical institutions may have seen the revised nurse staffing ratio as a kind of innovation. Therefore, the first-applied group in the initial stage of system introduction may have responded slowly to the revised system, and the second-applied group may have responded relatively quickly. Second, it has been shown that innovation and acceptance of new medical technologies are more active in medical institutions that are large, located in metropolitan areas, and where competition between institutions is fierce [31, 32]. The results of this study are in accordance with the diffusion theory of new medical technology, as the OR for nurse staffing ratio improvement was greater in institutions in metropolitan areas (second-applied group) with higher incomes of residents, a large number of physicians per unit population, and higher competition among medical institutions than in medical institutions located in rural areas and small- and medium-sized cities (first-applied group). As suggested by the diffusion theory of innovation for policies and new medical technology, it is necessary to acknowledge that some medical institutions may respond to the revised nurse staffing ratio with a delay or slowly.

### An approach to increasing nurse staffing

Although an incentive policy was implemented to encourage medical institutions to increase nurse staffing by revising the method of calculating the nurse staffing ratio, additional policies are still required to modify the behavior of medical institutions with low nurse staffing levels. First, it is important to assess whether the existing level of financial incentives is appropriate for medical institutions, and, if necessary, the level of incentives should be strengthened. If the incentive level is not high, medical institutions may not actively respond to the policy. Second, according to policy experiences and research findings on successful improvements in the nurse staffing levels in other countries, it is challenging to properly increase and sustain nurse staffing in medical institutions in vulnerable regions with only financial incentives [33, 34]. Support from the central or local governments is important for improvement in working and living conditions and the working environment, for granting and supporting opportunities for lifelong education, and for offering training and professional development. There is also a need to package and provide additional financial and non-financial incentives, such as improved supervision and management of nurses. Third, as this policy aimed to reduce the number of patients per nurse by increasing the absolute number of nurses, it is questionable whether it will increase the quality of nursing services. Increasing the number of nurses does not guarantee an improvement in the quality of nursing services. It is important to secure experienced nurses to improve the quality of nursing services. Several studies have consistently demonstrated the importance of experienced nurses [35]. Some policies in Korea also consider the proportion of experienced nurses a key indicator. For example, for medical institutions that provide hemodialysis, the evaluation criteria are not the number of nurses but the ratio of nurses with more than two years of experience in hemodialysis. The higher the number of nurses with more than two years of experience, the higher the quality of hemodialysis [2]. Therefore, to improve the quality of nursing services, a policy that goes beyond simply increasing the number of nurses should be considered and the proportion of experienced nurses should be included as a key indicator, so that the system can be improved in a way that secures more experienced nurses.

Finally, there is also an urgent need to legislate a new minimum nurse staffing level that all medical institutions must comply with. Due to the impact of the revised nurse staffing ratio, the number of grade 1 medical institutions increased to 32.1%, yet grade 1 standard (average of fewer than 2.5 patients per nurse) is the same as the minimum nurse staffing required by the Medical Act, which is 2 nurses per 5 inpatients. Nurses are legally required to work 40 h per week, take legal vacation time, and participate in education and training. If the value of 2.5 patients per nurse is converted into patients per nurse on duty, it becomes equal to the number of patients per nurse multiplied by 4.8 [36], which corresponds to 12. This amount is considerably greater than the number of patients per nurse on duty stipulated in other Organization for Economic Co-operation and Development (OECD) countries such as the United States and Australia. In Australia, even during night shift, which has the highest nurse-to- patient ratio, the number of patients per nurse is estimated to be 7–8 [37]. Therefore, it is necessary to lower the legal requirements for the number of patients to be cared for by one nurse and to elevate the standards for the nurse staffing ratio to the international level in order to reduce the excessive workload and exhaustion of nurses [10]. A strong regulatory policy is required to legislate a new minimum staffing level rather than practicing tolerance and imposing penalties on medical institutions. Several studies assessing changes after minimum staffing requirements were enacted by law in the U.S., Australia, and elsewhere, have demonstrated that enacting minimum staffing levels cost-effectively improves patient and nurse outcomes while closing the nurse staffing gap among medical institutions [4, 12, 38].

### Limitations

In this study, we did not measure the number of beds, patients, or nurses in each medical institution; instead, we analyzed changes in nurse staffing ratios based on the nurse staffing grade information disclosed on the HIRA website. Furthermore, changes in the nurse staffing ratios analyzed in this study do not necessarily indicate changes in actual nurse staffing levels. When the calculation method for the nurse staffing ratio is changed from the nurse-to-bed to the nurse-to-patient ratio, the nurse staffing grade may improve even if the number of nurses remains unchanged. The degree of improvement was greater in medical institutions with low bed utilization rates. The grade improvement effect caused by the elimination of unused beds in the nurse staffing ratio calculation would have resulted in rapid nurse staffing ratio improvement across nurse staffing grades 2–7, immediately following the implementation of the revised calculation method. However, the nurse staffing ratio graph of the prospective cohort (Fig. 1) and the ORs by year shown in Table 5 indicate that the improvement in nurse staffing ratio continued even after implementing the revised calculation method. Future research is needed to distinguish between the effects of removing unused beds and additional nurse staffing on improving nurse staffing ratios. Despite the limitations, this study is significant in that it established a prospective cohort of medical institutions from across the country to examine the effect of financial incentive policies on the improvement of nurse staffing ratios through the revised calculation method for the nurse staffing ratio by comparing the first-applied group, second-applied group, and non-applied group.

## Conclusions

After revising the calculation method for the nurse staffing ratio from the nurse-to-bed ratio to the nurse-to-patient ratio, there was a statistically significant difference between the first-applied group, second-applied group, and non-applied group in the timing and intensity of nurse staffing ratio changes. It was confirmed that the nurse staffing ratios of the medical institutions that implemented the revised nurse staffing ratio were significantly higher than those of the medical institutions that did not. Thus, it is necessary to expand the application target and scope of the revised calculation method (using the nurse-to-patient ratio) for the nurse staffing ratio to include all medical institutions nationwide, as well as general wards and intensive care units. It is necessary that all medical institutions, including those that do not respond or respond slowly to the revised nurse staffing ratio, should implement a policy that regulates the nurse staffing levels to elevate nurse staffing levels to international standards.

### Implications

Well-structured nursing policies can improve the output of nursing services by eliciting medical institutions’ voluntary participation; therefore, nursing policymakers must strive to establish successful nursing policies. This study demonstrates that financial-incentive policies can motivate healthcare organizations to hire nurses. For this policy to have a noticeable impact, its criteria should be realistic, and sufficient incentives should be provided. Additionally, it would be more effective to package financial and non-financial incentives to secure a sufficient nursing workforce. When implementing a policy, the target medical institutions should be given adequate time and compensation until they recognize and accept the policy change and modify their behavior. Furthermore, if the initial focus of the policy is on securing sufficient nursing staff, the next step should be on securing a large number of experienced nurses to ensure the quality of the nursing staff. Significantly, this will provide higher-quality nursing services and improve patient safety and healthcare performance.

## Data Availability

The research data is available upon request. To request the data, contact the corresponding author of the article.
